# Ethnokinesiology: towards a neuromechanical understanding of cultural differences in movement

**DOI:** 10.1098/rstb.2023.0485

**Published:** 2024-08-19

**Authors:** Lena H. Ting, Bryan Gick, Trisha M. Kesar, Jing Xu

**Affiliations:** ^1^ Coulter Department of Biomedical Engineering at Georgia Tech and Emory, Georgia Institute of Technology, Atlanta, GA 30332, USA; ^2^ Department of Rehabilitation Medicine, Division of Physical Therapy, Emory University, Atlanta, GA 30322, USA; ^3^ Department of Linguistics, The University British Columbia, Vancouver, BC V6T 1Z4, Canada; ^4^ Haskins Laboratories, Yale University, New Haven, CT 06520, USA; ^5^ Department of Kinesiology, The University of Georgia, Athens, GA 30602, USA

**Keywords:** motor accent, motor concept, individual differences, movement diversity, biomechanics, embodiment

## Abstract

Each individual’s movements are sculpted by constant interactions between sensorimotor and sociocultural factors. A theoretical framework grounded in motor control mechanisms articulating how sociocultural and biological signals converge to shape movement is currently missing. Here, we propose a framework for the emerging field of *ethnokinesiology* aiming to provide a conceptual space and vocabulary to help bring together researchers at this intersection. We offer a first-level schema for generating and testing hypotheses about cultural differences in movement to bridge gaps between the rich observations of cross-cultural movement variations and neurophysiological and biomechanical accounts of movement. We explicitly dissociate two interacting feedback loops that determine culturally relevant movement: one governing *sensorimotor tasks* regulated by neural signals internal to the body, the other governing *ecological tasks* generated through actions in the environment producing ecological consequences. A key idea is the emergence of individual-specific and culturally influenced *motor concepts* in the nervous system, low-dimensional functional mappings between sensorimotor and ecological task spaces. *Motor accents* arise from perceived differences in *motor concept topologies* across cultural contexts. We apply the framework to three examples: speech, gait and grasp. Finally, we discuss how ethnokinesiological studies may inform personalized motor skill training and rehabilitation, and challenges moving forward.

This article is part of the theme issue ‘Minds in movement: embodied cognition in the age of artificial intelligence’.

## 1. Introduction

Humans move their bodies for reasons ranging from social, practical, to survival, but we lack an integrated understanding of how culture and biology interact to shape the way we move. It is now recognized that humans are biocultural [1–4]. Our interactions with the environment—both physical and social—play crucial roles in sculpting our movements. Complex neural and biomechanical systems flexibly reconfigure our bodies to produce a wide variety of movements that are both physically and socioculturally useful. We can interact with the physical environment to move ourselves and other objects in the world, and we can also move to communicate through speech, body posture and gesture, both explicitly and implicitly. We use our movements to engage in social and cultural activities such as singing, dancing and sports to express our social and cultural affiliation. Yet, there remains a gap between bioscientific and sociocultural studies of human movement [1,4,5]. Separate research fields adopt different sets of conceptual vocabularies, theoretical frameworks and research methodologies, limiting progress of a coherent understanding of human movement. Even within these areas, there is little interaction between researchers focusing on different types of movement, e.g. studies of speech movement and dance movement remain in distinct fields, whether from a sensorimotor or sociocultural perspective. Some early thinkers called attention to the importance of considering both biology and culture in human movement. Mauss [6] famously coined the term ‘body techniques’ to emphasize the need to study cultural influences on the way people move. Higgins proposed that the structures of human movement are dynamic forms that emerge from both biological and environmental constraints [7]. Using concrete examples such as ‘stone knapping’ [5] and balancing [8], anthropologists have more carefully defined the biological and cultural constraints and their interactions that give rise to specific motor skills. In movement science, however, the absence of an explicit theoretical framework to study the mechanisms at this interface is conspicuous, despite detailed proposals by some early thinkers [9] and recent calls for bringing behaviour into neuroscience [10]. Building upon prior work at the movement–culture interface, we present a conceptual framework for the field of *ethnokinesiology* [11] to facilitate interdisciplinary research in identifying the mechanisms by which cultural differences in movements arise within and across individuals.

The variety of human movements resulting from culturally diverse ‘communities of practice’ has been well documented [12–14]. Linguistic and ethnographic studies highlight the diversity in human speech, postures and movements that can arise from frequent practices spanning from the mundane to the ritualistic. For example, our body postures in standing or sitting may vary depending on whether we habitually squat, kneel or sit in a chair in our daily routines [6,15]; similarly, even during non-speech, our vocal tract postures differ depending on what language we speak most often [16,17]; walking style may be influenced by the social and cultural practices we engage in, such as dance, martial arts, daily rituals or physical labour [5,8,18–22]; runners in cultures that prioritize running tend to adopt more optimal forms [23]; potters from different cultures use different finger and hand techniques to form similar pottery shapes [24]. At the neurobiological level, neuroplasticity underlies changes in neuromotor circuits in animals [25–27] and humans [28,29] shaped by behavioural repertoires. All these practices form a composite ‘accent’ in one’s daily actions parallel to an accent in one’s speech. At a population level, culturally shared movement practices may emerge, as in the many varieties of World Englishes [30] across different geolinguistic regions. A similar notion, ‘motor style,’ common to members of a given cultural group, has been proposed by Bril [5]. Here, we maintain a broad definition of *culture*, referring to common practices shared within a group as large as an ethnic group or a religion to as small as a high-school social in-group or a nuclear family. Expertise training provides some of the best-documented examples of cultural practices leading to biological structural changes [13]. Cortical sensorimotor areas [31] and subcortical white matter [32] have been shown to differ between experts and novices in various movement practices such as music [31] and sports [32]; spinal circuits also change with expertise in dance [33–35] and sports [36]. These studies reveal how cultural differences shape movement variances both within and across individuals. These differences are realized across ‘communities of practice’ [13,14] despite individual variations in implementation, as can be seen in kinematic and neuromechanical studies of gait; for example, individual-specific gait-like patterns of tongue movement have been shown to be associated with speech-rate ranges across speakers of the same language [37], and a large portion of individual differences in muscle coordination patterns for gait and balance have been demonstrated to persist across different biomechanical contexts and walking speeds [38–41]. Such individual differences in walking patterns can be identified through machine learning approaches [42,43] and persist over long periods of time [44], as well as across walking speeds [42,43,45]. Adopting principles and methodologies learned from movement studies of frequent practice and expertise, both within and across cultural communities of practice, can thus enrich and facilitate the study of ethnokinesiology.

Although ecological and environmental influences on movement are poorly studied, patient stakeholders (individuals with movement deficits and their caregivers) and healthcare clinicians commonly have therapeutic goals that encompass interactions of our body with the physical, social and cultural environment [46,47]. Bridging the gap between the biology and culture of movement is increasingly vital with the recognition that ‘movement is medicine,’ and with gait being considered the ‘sixth vital sign’ of health [48]. It is increasingly recognized that data-driven and biomedical studies within WEIRD populations (white, educated, industrialized, rich and democratic) may not generalize across ethnic, social or cultural groups [49–51]. While we typically think of ageing and disease as physiological phenomena, the interactions between biology and culture in human health are increasingly evident [1] and need to be understood. Biocultural interactions in movement are especially important in rehabilitation, where physiological injury or disease rarely correspond directly to movement impairments, and neuroplasticity is induced through movement practice [52]. Moreover, during clinical practice, when an individual with movement impairments owing to neurologic (e.g. Parkinson’s disease and stroke) or orthopaedic (e.g. wrist fracture and ankle sprain) pathology is asked to list their functional goals, these goals usually include interactions with their social environment: people with speech deficits state needs to communicate with their family and friends; people with lower-limb deficits would like to attend community social events or play soccer with their grandchildren; people with upper-limb deficits may desire to type or write again, cook and use utensils around their kitchen and dining table, or pick up their toddler [53–55]. For people with motor disabilities, primary motivators for showing up for physical or occupational therapy [55–57] may involve social (e.g. being able to walk to church or hold and hug their grandchild) and cultural factors (e.g. being able to kneel during prayer at a temple). Furthermore, social and cultural conformity play important roles in guiding an individual’s goals during rehabilitation. For example, individuals with amputations often place importance on cosmesis, sometimes to a greater or equal extent compared with function [58,59]; individuals with hemiparesis affecting one side of the body may exert a significant emphasis on the aesthetic symmetry of their gait [60]. From the developmental perspective, the cultural and social environment a child grows up in may also play a crucial role in the future treatment of motor impairments and rehabilitation training strategies.

Here, we provide a basic theoretical framework for understanding how both social and biological signals influence the *experience-dependent plasticity* of the nervous system [61] to shape individual and cultural differences in movement. In §2, we lay out the basic elements of the theoretical framework for movement given rise by dual feedback loops, representing both an external (ecological) loop in which task relevance is learned through information, including social signals from the outside world, and an internal (sensorimotor) loop, with signals arising from within the body as it moves owing to the biomechanical properties of the body defined by joints and muscles and calibrated based on physical forces such as gravity and inertia. This first-level schema can help generate and test hypotheses about cultural differences in movement to help bridge the gap between the rich observations of movement variation across cultures and the neurophysiological and biomechanical studies of movement. Both *ecological task* and *sensorimotor task* relevance can influence *experience-dependent neural plasticity* through reward- and error-based learning, leading to both structure and variability in movement execution. A key idea is the emergence of *motor concepts*, low-dimensional functional mappings between *sensorimotor task* and *ecological task* spaces that can differ across cultures and practices, providing a mechanistic foundation for the formation of *motor accents* [39]. In §3, we use our dual ecological and sensorimotor feedback framework to interpret individual- and practice-specific differences, and how the same framework can be extended to account for cultural variations. In §4, we discuss how advancing the mechanistic understanding of individual and cultural differences in movement will have practical applications in personalized approaches to motor skill learning and rehabilitation that optimize mobility in health and disease. Finally, in §5, we identify theoretical and methodological challenges for future studies in ethnokinesiology, which will require leveraging modern technologies in motion recording in the lab as well as ‘in the wild’ and their interpretation based on both data-driven and biophysical modelling approaches. We discuss how different levels of investigation across fields and cross-fertilization in the context of a unified theoretical framework can advance the study of ethnokinesiology.

## 2. A basic theoretical framework for ethnokinesiology

**Figure 1.**
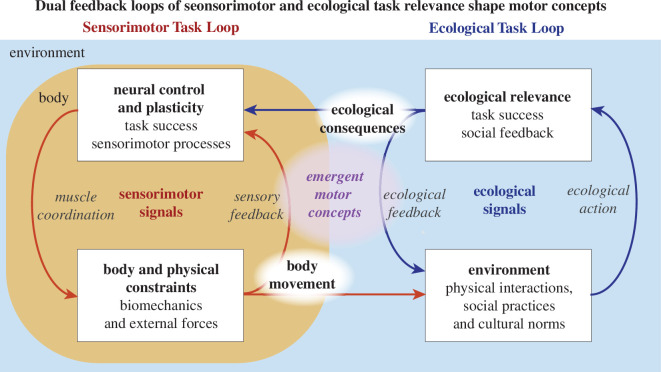
A conceptual framework for guiding ethnokinesiology studies. This first-level schema explicitly dissociates the role of sensorimotor task and ecological task feedback loops in experience-dependent neural plasticity. The interactions between these two loops result in the emergence of a set of ‘motor concepts’ that map sensorimotor tasks to ecological task spaces that can differ across cultures and practices. The emergence of motor concepts leads to both structure and variability in movement that are sculpted by both the physical and cultural environments in which we move.

In this section, we describe a theoretical framework for ethnokinesiology in which interacting *ecological* and *sensorimotor task loops* give rise to encultured movements (figure 1). The bridging of cultural and biological processes to facilitate the understanding of how culture is embodied has been proposed by biocultural anthropologists [5,8]. Yet, it is not clear *how* these two processes interact to create cultural differences in the neural control of movement. The body and its nervous system live within the context of both physical and social environments and are shaped by behaviour [10,62]. Here, we dissociate the feedback loop residing in the internal sensorimotor processes (figure 1, orange box) that has been the focus of many neurophysiological studies of movement, from the ecological feedback loop that includes sociocultural influences as described by many ethnographic accounts (figure 1, blue box). The sensorimotor loop encompasses processes internal to the body such as neural signals arising from sensory receptors, neural computations and ultimately motor signals to the muscles (figure 1, red arrows); both are physically constrained by the biomechanical properties of the body and by forces such as inertia and gravity. Once a motion occurs, its interactions with the environment provide *ecological signals* that are used by the nervous system to attach meaning to a movement that may be physical, social or both (figure 1, blue arrows). Reward and error signals regarding the *ecological actions* generated by a movement, such as touching an object or eliciting a smile, then shape the sensorimotor processes giving rise to future movements.

The relationships between *sensorimotor* and *ecological signals* are often implicit, and not explicitly addressed or dissociated in research studies. Most neuroscientific studies focus almost entirely on either the *sensorimotor loop* or the *ecological loop*. For example, many motor control studies are restricted within the sensorimotor space (figure 1, orange box), where precise sensory and motor signals (figure 1, red arrows) are studied within the context of highly specified tasks, such as walking [10]. In these studies, there may be little variation of the ecological feedback loop from the physical environment, and social feedback is typically strictly controlled and unaccounted for (although it can be an important factor in human movement studies, e.g. the ‘white coat syndrome’ [63]). Other motor control studies are confined within the ecological space (figure 1, blue box), measuring only the resulting movements and whether they accomplish *ecological tasks* successfully such as hitting targets or producing intelligible speech (figure 1, blue arrows), as an indirect way to infer internal sensorimotor processes (e.g. [64]). However, to identify causes of individual and cultural differences in movements, it is necessary to consider how the ecological feedback from diverse behaviours affects sensorimotor control of movements, and specifically how building blocks of movement can differ across individuals owing to diverse ecological factors. Here, we present the idea that the nervous system forms *motor concepts* (figure 1, purple bubble) to facilitate coordinating multifunctional and complex body parts to achieve ecologically relevant outcomes. *Motor concepts* are low-dimensional functional mappings between *sensorimotor tasks and ecological tasks* that can differ across cultures and practices. Perhaps the most salient example is the production of basic speech sounds across languages; well-known cultural differences in speech sound production and perception may be studied in terms of language-specific distinctive movement combinations or phones [65–67], but seldom with regard to how they can be generated by the neuromuscular system [68–70]. These explicit *phones* are examples of culturally defined *motor concepts* that are composed in combinations of multiple processes to form meaningful words and sentences for communication. While this basic schema is not intended to be comprehensive, it provides a simple and tractable model that explicitly addresses the need to learn mappings between internal biological processes and physical–social interactions in the environment, thus providing a mechanistic foundation for the formation of speech accents, and more generally *motor accents* [39].

The *sensorimotor loop* internal to the body learns the motor and sensory signals required to coordinate the body. Consider the ‘motor babbling’ [71–77] of a baby as it learns to move through sensorimotor exploration that is refined through interactions with the world. Motor babbling may not accomplish ecologically relevant outcomes (figure 1, blue box), but it is a discovery process to uncover and train the sensorimotor loop (figure 1, orange box) to create patterns of movement afforded by the biomechanical constraints [78,79]. Neural signals activating muscles (figure 1, red descending arrow) generate body movements (figure 1, bottom white box) that cause sensory signals from the movements to be returned to the central nervous system (figure 1, red ascending arrow). A set of body motions resulting from the balance of physical forces generated by the body, gravity and physical environment, selected and calibrated only by internal sensory feedback (figure 1, red ascending arrow) must be learned. A *sensorimotor task* thus enables the coordinated actions of the body necessary to support potentially useful movement, such as extending and flexing a leg, shaping a facial configuration or positioning the fingers. Each *sensorimotor task* takes advantage of the multifunctionality and reconfigurability of the body for moving in a multitude of ways, overcoming the ‘curse of dimensionality’ problem [80] that must be confronted. The establishment of learned, habitual movement patterns that may be reinforced by evolutionary and genetic mechanisms enables the body to establish a limited repertoire of *sensorimotor tasks* from among the many possible. These tasks are often manifest in studies of motion primitives [81–83] (at the level of force and movement) and of muscle synergies or motor modules [39,81,84–87] (at the level of motor neuron and muscle activity patterns). The vast redundancy of the musculoskeletal system enables countless different patterns of muscle activity to be ‘good enough’ solutions for many different *sensorimotor tasks* [39,88].

A movement is attached to ecological meaning once a real-world consequence is obtained. When a movement generated from a *sensorimotor task* is executed in and with awareness of an ecological environment (figure 1, red arrow from orange to blue box), it will produce a socially and physically relevant *ecological action* (figure 1, blue ascending arrow), where reward and error signals owing to *ecological consequences* (figure 1, blue arrow from blue to orange box) shape the sensorimotor control of movement. As such, the learned sensorimotor task supports successful exportation to enable reward-based or reinforcement learning [89–92]. Consider again the motor babbling of a baby, where some of the movements may cause physical *ecological feedback* (figure 1, descending blue arrow) resulting from touching an object, bringing food to the mouth, or moving the body from one location to another. Yet, the earliest *ecological feedback* of a movement may also be from social interactions owing to its interpretation by others. Smiling or making sounds such as ‘da’ or ‘ma’ may result in positive attention from parents, and pointing may cause others to act, e.g. by bringing food or objects. At the same time, *ecological consequences* of a movement are sent back to the agent (figure 1, horizontal blue arrow). Reward and error signals then reinforce the sensory and motor signals that generated the successful *ecological actions* (figure 1, blue ascending arrow), establishing relevant *sensorimotor tasks* that confer utility. Owing to experience-dependent neural plasticity, sensorimotor circuitry is shaped continuously through the ‘practice’ of daily life, both physical and social. Motor babbling can bias individual differences [93] in sensorimotor control based on which muscle activation patterns are first recognized as generating a successful *ecological consequence* [88], which will be repeated and refined; a similar idea can be applied to habit formation [94]. Cultural differences in movement can also be embodied by the same principle. Physical environments can be culture-specific, shaping the particular *sensorimotor tasks* that hold ecological relevance. Consider manipulating food using one versus two hands, chopsticks or forks and knives, or differences in terrain and dwelling styles that affect whole-body coordination required for gait and balance. Moreover, every culture imposes a certain ‘standard’ for a member to carry out one’s body movements in various social interactions, such as greeting, walking, sitting and eating manners. The ways we gesture or manipulate food with our hands or utensils are all associated with cultural norms [95]. People within each group will often implicitly imitate, and sometimes magnify, the unique features within the group to enhance their membership identity. Indeed, two of the authors have had the personal experience of being recognized as outsiders when walking among racially, but not culturally, similar groups. Aesthetic preferences that differ across cultures, such as in singing, dance or social communication can also shape postures and gestures within groups and sub-groups. As such, the same *ecological task* can be achieved by multiple *sensorimotor tasks* depending on social objectives.

A *motor concept* is an emergent set of relations between a ‘real-world’ *ecological task* and an embodied *sensorimotor task* via a movement and its experienced (direct and indirect) *ecological consequences*. A *motor concept* (figure 1, purple bubble) emerges through the repeated experience of associating a bodily coordination pattern with a corresponding set of external (social, cultural, and physical) *ecological consequences*. Here, we follow ideas from psychology where cognitive concepts form a basis for a theory of cultural differences in neural processes. Eleanor Rosch has famously suggested that ‘concepts are the natural bridge between mind and world’ [96]. While there are symbolic (linguistic) aspects of concepts, Rosch emphasized the continuity between the mind and the world, the inseparability of the organism (body and behaviour) and the environment [96]. The most familiar cultural differences in how we move are the vast variations of movement creating speech sounds across languages that underlie accents in how we talk. The same principles can apply to movements in the absence of conscious awareness or symbolic representations, as we learn to walk, manipulate objects, and perform other motor tasks. *Motor concepts* are formed following principles similar to those described by Rosch, requiring constant interactions between the physical body and the environment. As such, the *motor concept* distinguishes an ecologically relevant movement from a non-ecological or pre-ecological movement seen in motor babbling (e.g. [97]). However, they do not directly represent the external world, but rather need to exert meaningful actions in the cultural and physical worlds using the biological sensorimotor system [98–101]. *Motor concepts* such as ‘kicking a ball’ or ‘holding a fork’ may be shaped explicitly and implicitly in sports, dance, music and other rituals. *Motor concepts* can also be transmitted socially through cultural evolution, in which behavioural regularities facilitate communication and consolidation of group identity [4,102], regularities that have been shown to disappear within two generations in immigrant groups [103].

**Figure 2.**
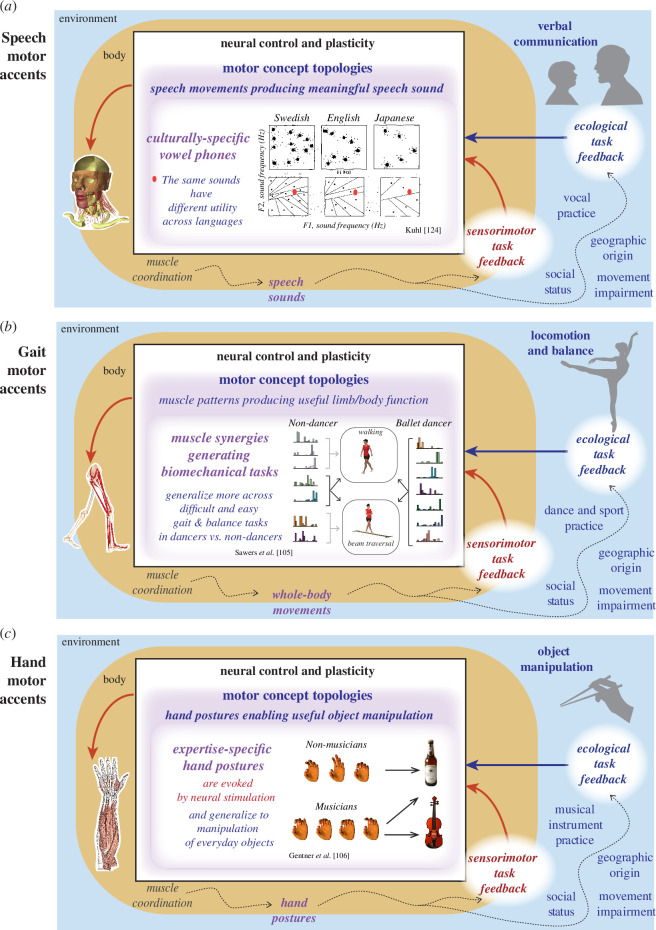
Motor concept topologies for speech, gait, and grasp underlying the formation of motor accents. Common across these examples is the idea that motor concept topologies are different across cultural subgroups, leading to motor accents when applying one set of motor concepts in another context. (*a*) [104] In speech, the movements that generate vowel phones differ across cultures, with each language defining a different number and distribution of vowel phones used to produce speech. A speech motor accent arises when the motor concept topology of phones from one language is used to map motor concepts to another language. (*b*) [105]. In gait, motor concepts are associated with muscle coordination patterns called muscle synergies that are consistently used by an individual across different gait and balance tasks. The generalization of muscle synergies that are sculpted by ballet dancing across both a difficult beam traversal task and overground walking provides an example of how motor accents can arise from practising specific types of movement. Implicit practice of culturally relevant movements could similarly create motor accents in gait. (*c*) [106]. Evidence of training-based neural circuit changes is revealed by hand postures elicited through stimulation of the motor cortex, which differ in musicians and non-musicians. Hand postures create useful motor concepts for object manipulation that differ from musicians who play instruments versus nonmusicians. Similar to gait, the specific training of the musicians also influences their grasping behaviour in everyday life, as shown in their multi-joint coordination. Figures adapted with permission from [105–107].

*Motor accents* are variations in motor style that result from *motor concepts* varying in number and structure across cultures and movement practices. Here, we define *motor accents* as the systematic variations in stereotyped and predictable movement patterns that are characteristic of an individual or a subgroup in a society. As discussed above, an individual’s prior experiences shape their *motor concepts*, as well as the ways in which the associated *sensorimotor tasks* are performed, based on *ecological consequences*. A learner within a specific ecological environment forms their *motor concepts* in alignment with cultural norms, creating a culturally influenced, yet individual-specific, *conceptual topology* (figure 2, purple). In speech, these topologies are formed based on the phones, such as vowel sounds, that must be distinguished in a particular language; in walking, a motor concept topology could be related to how one places the foot, supports bodyweight, propels the body forward, lifts or swings their leg; in grasp, such topology could correspond to an inventory of habitual hand postures; we will discuss these examples in detail in the following section. *Motor accents* become evident when a learner moves from one environment to another, such that their existing conceptual topology becomes superimposed onto the topology of a new environment, leading to a perceived misalignment relative to cultural insiders. Analogous to a speech accent, when one encounters a new movement, it will first be constructed by using one’s existing set of *motor concepts*, which may have different boundaries and prototypes across one’s experience and cultures [108–111], producing predictable biases or deviations in how the movements unfold based on their existing priors [110]. These systematic differences allow us to identify others by their movements, and to make (often stereotyped) judgements about their culture, such as social status, country of origin or sexual orientation [6,22,112]. Motor accents thus only exist on the observer’s end in a cross-cultural context, as our perceptual system may also be shaped through similar mechanisms [108,109].

## 3. Cultural differences in motor concepts underlie motor accents in speech, gait and grasp

In this section, we show how this common theoretical framework for ethnokinesiology can account for how motor accents arise across divergent motor behaviours. Speech, gait and grasp are examples of motor behaviours that use different parts of the body with different neuromotor constraints, including divergent interactions with both physical environmental forces and cultural systems. Using the common principle of *motor concepts* to provide a bridge between sensorimotor and ecological tasks can help reveal parallels across seemingly divergent motor behaviours and provide a roadmap for progress in ethnokinesiology. First, we use the proposed frameworks to detail the convergence of *sensorimotor* and *ecological task loops* in speech that underlie the formation of speech accents based on *motor concepts* that define meaningful sounds within a culture. For most other bodily movements, e.g. gait and grasp, such explicitly defined *motor concepts* owing to cultural practices have not been quantified. However, modular neural structures for control of movements refined and modified by specific practices such as dance [105,113] and music performance [106] have been shown. While these studies were originally intended to show the effects of motor expertise on neural plastic changes to support motor skill, much of the literature on biocultural anthropology is focused on highly skilled movements in culturally specific dance, ritual or other movement practices [4,6]. These highly practised and skilled individuals provide an illustration of the extreme capabilities of humans to control their bodies in different ways, revealing fundamental neural plasticity and motor control principles. We argue that the same biocultural processes underlying movement such as speaking, walking and manipulating objects can give rise to consistent cultural biases in movements through the ‘patterned practice’ [12] of everyday life. By couching each example within the conceptual framework (figures 1 and 2), we hope to offer some examples of ways in which ethnokinesiology may reveal how and why cultural differences in movement arise.

### (a) Speech

Although speech is one of the most defining—and most studied—culturally diverse aspects of human behaviour, speech as a system of movement has received comparatively little study. Speech is inherently a cultural phenomenon, and we are all familiar with speech accents produced by non-native speakers learning a new language. Yet, it is not well understood *how* these differences arise from a sensorimotor perspective. Speech is often counted among nature’s most complex movement systems, generating dozens of discrete, meaningful movements per second [114] that combine to communicate an unlimited variety of messages with a subtlety and precision available only to the human species. Babies are born with *sensorimotor* mechanisms capable of producing many of the basic movements that later appear in speech [97], even in extreme instances of anencephalic neonates who lack intact brain structures above the brainstem [115]. As with other movements, we learn to use the body to produce speech through experimentation that starts in the womb, which enables the nervous system to discover how to take advantage of the biomechanical properties of the body [79]. In the vocal tract, some of these biomechanical affordances constrain the kinds of movements reinforced by the *sensorimotor feedback loop* that later become speech movements [78,79] and continue to shape the movements of human speech into adulthood [68,116]. Of course, prior to exposure to the world outside the womb, these movements lack social meaning.

Oral movements become meaningful and culturally differentiated through interactions with the physical and social environment. As babies are born, grow and continue to experiment with the movement capacities of the vocal tract, they quickly discover how their movements interact with the world, sometimes generating familiar or imitative sounds, and sometimes eliciting positive social responses that reinforce those movements. Through repeated exposure to their *ecological consequences*, our initially stereotypic speech movements are updated to become increasingly ecologically relevant. In our proposed model, this growing understanding of the relationship between a movement and its *ecological consequences* is at the core of the emerging *motor concept* (see figure 1 above). In the case of early oral movements, a baby capable of phonating during its first 1–2 months after birth may learn through repeated experimentation that it can consistently modulate its vocalization with, e.g. lip-rounding, by the end of its first several months (e.g. [117,118]); through this repetition, in the present model, the baby develops the ability to perform a sensorimotor task, initially producing the movement via a self-reinforcing feedback loop (e.g.: ‘I did that!’ or ‘I can do that again!’). If the movement elicits a positive response from, say, a caregiver, the movement is both reinforced and acquires the beginnings of an association with some aspect or property of the environment, which the present model characterizes as an *ecological task*. Initially, the *ecological task* that is helping to reinforce the movement may be as simple as: ‘Make a familiar sound,’ or ‘Get Mom to look at me.’ However, as the baby builds a larger repertoire of movements through the remainder of their first year (e.g. [117–119]), these associations differentiate into a growing ‘vocabulary’ of response-generating movements, tuned to elicit increasingly differentiated social responses based on the language and culture of its surroundings, hence reinforcing cultural differences. A ‘speech sound’ is an example of a *motor concept* that emerges through repeated experience of associating a stable oral coordination pattern with a corresponding set of social–ecological consequences that differ across cultures.

Ecologically whole, potentially meaningful ‘units’ of movement—what linguists would normally call ‘phones’—are one scale of *motor concepts*, composed in combinations of multiple processes to form meaningful words and sentences for communication. Below the level of the phone, some speech movements may correspond in size to discrete body movements sometimes called ‘gestures,’ described by Browman and Goldstein [120, p. 156] as abstract ‘characterizations of discrete, physically real events that unfold during the speech production process.’ At least some sub-phone speech gestures may correspond to the embodied movements (rather than abstract model characterizations of movements) that Gick *et al*. [68, p. 835] describe as modular structures ‘associated with different muscle groupings, suggesting separate motor modules underpinning their control,’ and later (p. 840) as ‘the primitive units of speech motor organization;’ embodied units of this kind have also been referred to as ‘devices’ [69] or ‘articulators’ [70] that reconfigure the body to perform different tasks. Above the level of phones, larger motor concept ‘chunks’ (such as syllables, e.g. [121]) can emerge at any scale actionable by the body and its systems (*sensorimotor tasks*) that correspond to useful *ecological tasks*. Thus, at a variety of ecological scales, *motor concepts* may emerge as elements of complex speech movement sequences.

With continued practice, speech movements become distributed within a culturally characteristic *topology* in the sensory–ecological functional space that linguists commonly refer to as the ‘phonetic inventory’ of a particular language. One common way to talk about such a topology for a subset of speech movements is in terms of an acoustic ‘vowel space,’ often used as a proxy for both perceptual and movement parameters (e.g. [122]). A vowel space specifies a low-dimensional mapping for a subset of vocalic movements in a language, generally excluding most consonants and many non-resonance characteristics of vowels (e.g. tone, voice quality, etc.). A typical vowel space for a language is characterized according to relative values of the first two acoustic resonances or formants (figure 2*a*, F1 and F2 for three languages), which are determined by vocal tract shape [123]. Topologies in vowel space vary largely according to how many distinct vowels a particular language has, which determines how densely packed the motor–conceptual space is for that language, a distribution (or ‘dispersion’) to ensure contrasts among the *motor concepts* that populate the space [124]. The number of distinctive vowel phones differs dramatically across languages, with at least 13 in Swedish, 8 in English and 5 in Japanese [104] (figure 2*a*). Thus, the same objective space in terms of auditory properties may be topologized in different ways, such that the same sound may be mapped onto different phones for speakers of different languages (figure 2*a*). Babies start as universal language learners when they begin babbling [125], and a baby’s native language environment shapes their mother tongue by forming perceptual biases towards a particular phone topology, which, in turn, drives culturally specific speech production [107]. Emphasizing the role of social interaction, foreign language learning in babies is more effective when they are instructed by a person rather than when watching a video of the same content, suggesting an important role of social reward and perceptual–motor interaction [107].

The topological distribution of *motor concepts* within a particular cultural ecological functional space determines a person’s ‘*motor accent*’ as perceived by a native speaker listening to a non-native speaker [126]. Learning an unfamiliar language requires the learning of new culturally defined *motor concepts* using the same biomechanical system. The topology of speech movements learned for one language will typically be an ill fit to that of a new language (figure 2*a*, red dots). Some of the existing *motor concepts* may export tolerably well to the new topology while others can create stubborn challenges. A speech accent can be viewed as a consistent, culturally dependent bias or error in movement generated when the *motor concept topology* of the native language is applied to a new language, by mapping the nearest *motor concepts*, or phones, from the native language to the new one. Distinct accents can also occur between culturally distinct social groups speaking the same language, and can incur heavy social costs (e.g. [127,128]), occasionally even with life-or-death consequences, as in the biblical accent distinction in which pronunciation of the word ‘shibboleth’ was used to distinguish in- versus out-group members [129]. While the objective set of frequencies representing the phones may overlap, a non-native speaker will tend to produce it as an example of a phone in their own language, which may or may not constitute a good match to that of the new language. The density of the conceptual maps also shapes our perceptual–motor coupling such that we are more likely to perceptually distinguish phones that have counterparts in our native language. This perceptual–motor coupling may underlie the common anecdotal observation that we tend to have relatively poor awareness of our own accents when speaking a second language [126]. In some cases, two distinct phones in the topology of one language could be mapped to a single phone in another (figure 2*a*), rendering the two sounds indistinct for speakers of the second language. A well-documented example of this is the observation that many Japanese speakers have difficulty in distinguishing and producing English /r/ and /l/ sounds [130]. As the proposed model shows that our own social movements are trained in large part on sensory (auditory and visual) information about the movements not just of ourselves, but of others, it predicts that, at least for movements learned in social–ecological spaces, our estimations of our own movements—our internal models [131]—particularly when interacting cross-culturally, may be inaccurate.

### (b) Gait

There are well-established accounts of cultural differences in gait and whole-body movements across social groups as well as physiological changes occurring in practitioners of culturally specific rituals and movements [19]. We all have had the experience of recognizing someone at a distance by the way they walk [132] or intuited that someone is a dancer or participates in a specific sport through the quality of their gait. Bril [5] proposed that ‘motor styles’ can be attributed in part to systematic cultural differences by more precisely assessing motor patterns using kinesiological analyses. Such an approach could lead to a neuroscientific explanation of the differences in gait that Mauss [6] noted in the walking characteristics of American versus French children, or those raised in a convent. He additionally noted that differences in movement techniques are a matter of culturally specific aesthetics and practice owing to culturally specific ‘bodily games’ such as those involved in dance, work and rituals. There is evidence of gross physical anatomical changes owing to cultural practices such as squatting or kneeling owing to use-dependent remodelling of the musculoskeletal system [133]. Beyond physiological changes to the body, there is also evidence of neural plasticity in sensorimotor systems underlying practices such as in Brazilian capoeira [8] or freediving [13] practices across multiple cultures. The accompanying adaptations in the processing of sensory information or regulation of the body functions are extreme examples of how task-specific neural plasticity can occur. However, neuroplastic changes can also be induced through the practice of more subtle, yet culturally important, differences. For example, social status and sexual orientation may often be detected based on gait parameters, e.g. ‘gaydar’ [112], often at great social cost [22]. Instructing people to walk with gender-typical versus gender-atypical gait patterns affects the accuracy and sensitivity of observers’ perceptions about an individual’s sexual orientation and gendered body motions [134]. Such differences are more likely to be determined through social–ecological feedback versus defined by physical constraints of the body or environment. However, subtle, yet perceivable gait differences across cultures are only beginning to be studied and quantified through objective measures [20,42,43].

Walking is a form of gait requiring sensorimotor coordination of the body to achieve efficient energy exchange for forward motion that can differ across cultural groups. In locomotion, the body’s limbs and trunk must be coordinated such that they can generate propulsive forces that can move the body from one location to another. The ecological task of locomoting can be accomplished by moving the body in many ways, such as crawling, skipping, hobbling, hopping, or running. However, walking leverages mechanical energy transfer as the body acts as a spring-loaded inverted pendulum, making it an energetically efficient form of gait [135–137]. The limbs are coordinated by the nervous system in walking to generate the biomechanical sub-tasks corresponding to the *motor concepts* of stance control, postural stability, limb advancement and propulsion. Yet, these strong biomechanical constraints are insufficient to uniquely determine muscle coordination for a given trajectory of joint angles and joint torques during walking [138], leaving ample room for variations based on culture and practice. To walk efficiently, the body should move as an inverted pendulum with as little muscle effort as possible, taking advantage of the passive dynamic properties of the body [136,139]. However, within the manifold of possible muscle activation patterns to perform a biomechanical task—also referred to as the ‘null space’—[138,140,141], there can be variations that confer social or cultural importance without increasing energetic expenditure substantially. For example, African women who are proficient in load-carrying are more energetically efficient than Western soldiers when loaded but not when unloaded because they are better able to coordinate their bodies to leverage pendular dynamics when carrying loads [142,143]. Nepalese porters also have greater load-carrying efficiency, for which different movement strategies have been hypothesized [5]. Here, we posit that the sensorimotor mechanisms underlying the production of *motor concepts*, as well as the structures of *motor concept topology,* could underlie aspects of cultural differences in gait.

As in speech movements, *motor concepts* for gait are learned and refined in our social and physical interactions. Babies are born with the ability to perform the *sensorimotor tasks* of leg flexion and extension [79,144], but these do not yet accomplish *ecological tasks* such as weight support and propulsion needed to walk [90]. Through physical and social interactions with the environment, babies learn through reinforcement learning to use their bodies to sit, crawl, stand, and walk. The initial forms of locomotion used by babies can be quite divergent because the biomechanical constraints are less stringent than those for a walking gait. Through motor exploration, a baby may learn to scoot themselves with their legs or crawl to locomote, using an extant mechanism for executing a *sensorimotor task* to achieve an *ecological task*, leading to the further refinement of the motor concept of propulsion. These early forms of locomotion, as well as early walking patterns in toddlers, do not use adult-like mechanical energy exchange mechanisms and are thus energetically expensive [145]. Social feedback also plays a role in the refinement of kicking from a pre-ecological *sensorimotor task* into a useful, ecologically relevant task, which can be enhanced through encouragement [91]. Ultimately, both physical and social feedback shape how babies learn to walk. As walking is inherently unstable, it is important to develop a *motor concept* to avoid falling. Cultural differences in diapering and swaddling also affect how and when children learn to walk [146] and they likely play a role in shaping their *motor concepts* for gait. The development of walking behaviours may also have strong social pressures as children strive to mimic their parents [90,91,145], and as such the learned behaviours may further take on characteristics of movement that are not necessary to perform the simple physical *ecological task* of moving from one location to another. Whereas speech sounds are enabled by biomechanics and constrained primarily by cultural factors and constraints on gait are largely biomechanical, social and cultural task relevance can still shape individual variations in accomplishing the physical ecological task. Furthermore, in contrast to speech sounds, cultural influences may be neither symbolic nor conscious, and *motor accents* in gait may result from implicit or explicit mechanisms for imitation [147] as well as a desire to conform socially.

Muscle synergies reflect sensorimotor coordination patterns supporting the performance of *motor concepts*, which may underlie culture-specific motor accents. Muscle activation patterns during gait can be decomposed into discrete patterns of muscle coordination referred to as *motor modules* or *muscle synergies*, which define proportions of synchronous activation of muscles that can robustly produce specific biomechanical actions [40,148,149] (figure 2*b*). Muscle synergies reflect the sensorimotor processes within the nervous system that implement a *motor concept* and are modulated over time according to the demands of the *ecological task* [150,151]. *Motor concepts* may underlie what we colloquially refer to as ‘muscle memory,’ but they reside within the nervous system [39,152]. Moreover, the *motor concepts* themselves are not uniquely determined by the biomechanical constraints, as these concepts could change after training, injury, or disease, e.g. limping. As children develop, the number of muscle synergies may increase from two to three and eventually to four or more, depending on the sampling of muscles measured [39,144,153]. In adults, the number and structure of muscle synergies are specific to an individual and used to construct walking movements across speeds [41,153], or even to recover balance during walking and standing [38,41,153–155]. In ballet dancers, an increased number of muscle synergies can be found during walking [113] that are used both in nominal overground walking and when traversing a narrow beam. In contrast, non-dancers used a smaller number of muscle synergies in walking and beam traversal, with only two being common across tasks [105]. These results suggest that ballet training may increase and refine the set of *motor concepts* and their *topological* relations that could result in a recognizable *motor accent* during walking. Moreover, muscle synergies supporting these *motor concepts* in ballet dancers are likely more energetically efficient, using more precise activation of muscles and less energetically costly muscle coactivation seen in both healthy and impaired populations [40,153,154]. A similar principle could be at work in African and Nepalese load carrying, with future studies yet to be done at this level of investigation. Differences in *motor concept topologies* could also be formed unconsciously through cultural and social feedback or culturally specific practices such as hunting, dancing, bowing, load-carrying, or walking in different types of footwear, creating recognizable motor accents in gait. To date, only a few quantitative studies have demonstrated gait biomechanics differences between Western and Eastern countries, as well as those arising from variations in other cognitive, racial [156] and cultural factors [18,19]. These studies also lack measures of muscle activation necessary to identify the underlying neural mechanisms that may be at play in coordinating the body’s biomechanical interactions across environments. Encultured practices such as dance and other ritualized movements may provide a good entry point for studying the role of socia–ecological factors on gait and movement [8], as they also offer a more direct way to understand and identify *motor concepts* for gait and other movements that may not be well-defined at either the ecological or sensorimotor level.

### (c) Grasp

The human hand presents an enormous movement repertoire that enables huge variances across individuals, social groups, and cultures. Hands are highly flexible in their function owing to their large number of biomechanical degrees of freedom [157–159] and their vast cortical structures and descending neural pathways [160–162]. This extensive flexibility allows the human hand to explore very large movement spaces to form new motor repertoires and to improvise solutions in novel situations. A wide variety of motor solutions using the hand can achieve the same basic functions, such as grasping an object or pressing a key. Different social norms and practices thus have a sizable ‘null space’ in which to impose and canonize their distinct features to enhance group identities, resulting in tremendously rich cross-cultural and subcultural variabilities. The diverse hand techniques used to form the same pottery shapes across different cultures illustrate how cultural variances of motor repertoires achieve similar functions [24]. Hand postures used to grasp a teacup also vary greatly across different cultures, e.g. between British and Japanese tea etiquettes. At the more expressive level, hand postures in dances, such as mudras in Asian dances across multiple cultures, are meticulously designed to carry important symbolic meanings. Cultural practices in everyday life also lead to different emphases on which hand to use. Contrary to the prevailing picture of human handedness with 90 : 10 right–left ratio [163], analysis of films among three traditional cultures in Africa and South America revealed consistent but rather weak right-handedness (~55 : 45) [164]. The two hands were close to being equally divided among non-tool-use tasks, and right-handedness only emerged in tool-use tasks (~84%), especially when precision grips were involved. Similarly, tasks involving precision grips are predominantly right-handed [165] and thumb–finger opposition tasks present higher finger independence in the dominant (right) hand [166,167]. In non-industrial societies, the pressure of using precision control with hand-held tools is relatively weaker (e.g.~15% among all the filmed activities in [164]), which may explain the relatively lower percentage of righthandedness compared with the Western standard. Handedness across cultures and its relation to tool use and precision control thus illuminate the intricate interplay between the biomechanical and neural constraints versus social and cultural influences on hand use in tasks as simple as grasping.

Within our theoretical framework, individual- and cultural-specific hand *motor concepts* are shaped through constant training of the *sensorimotor task loop* by the *ecological task loop* attaching *ecological consequences* to movements. Just as with the vocal tracts and legs, babies exhibit spontaneous hand movements even starting from when they are in the womb, from the palmar reflex to scratching and thumb sucking, to hand-to-mouth, hand-to-face, and hand-to-head movements [168]. Newborns use their primitive palmar reflexes to grasp anything placed in their palms. Soon they start to explore the world around them using their whole hands and, later, single and combinations of fingers [169–171], and the development of dynamic control of fingertip forces extends well into adolescence [172]. Discrete spontaneous hand postures appear months before socially meaningful gestures [173], and it is suggested that these movements are later recruited to serve specific functions [79,174]. Within our theoretical framework, these new hand and finger movements are *sensorimotor tasks*, shaped by the *sensorimotor task* loop during motor babbling, when a baby is exploring all possible body movements. When a baby attempts to make things happen in the real world, such as grasping a bottle or pushing on a toy button, these movements constitute *ecological actions*; success may be stumbled upon with a certain configuration of their hand and fingers, e.g. when the bottle is secured or when the music is played, the action will provide feedback to the body as *ecological consequences*, and hence be reinforced and repeated, and eventually incorporated into the motor repertoire. Just like a baby learning to build their new motor repertoire, the *ecological* and *sensorimotor task loops* happen in parallel when a novice tries to learn a new motor skill, such as picking up guitar playing. Motor babbling in learning a new musical instrument, sometimes referred to as unsupervised learning [175], allows the novice to explore possible new movements that their body is capable of making. Explicit external ecological feedback also plays a critical role through coaching and audience responses. Once selected, movements are honed by repetition and error-based learning.

Hand usage in expertise is an example of complex hand *motor concepts* and *topologies* formed through interactions between *ecological* and *sensorimotor task loops*. During development and through learning, simpler units of *motor concepts* in their basic forms are refined and new concepts are formed by exploring boundaries of biological constraints. More complex *motor concepts* can also be formed by superimposing or chunking together simpler units, and ultimately forming a hand-usage *motor concept topology* unique to each individual. The ecological settings where these refining and chunking processes occur play an essential role in coaching and sculpting individually unique *motor concept topologies*. While right-handedness appears to be result from the pressure of tool use and the demand for precision in the task [164], at the extreme of precision control, proficiency in high-level manual skills requires extensive practice, such as in professional musicians, who spend thousands of hours practising their hand skills [176]. This extensive practice pushes the boundaries of their biomechanical constraints, such as increasing the finger individuation abilities among pianists [177,178] by reducing the unintended activity of other fingers. Like cultural variance, experts in sports and music performance develop their own sub-sets and sub-cultures of unique hand-use *motor concepts*. For example, baseball and American football players have developed distinct hand shapes and throwing techniques for ball throwing owing to the different shapes of the balls and the activities of the throwers and receivers [179]; fingering techniques on a cello are very different from those on a violin, owing to the different shapes of the fingerboards and body postures of the musician [180].

*Motor accent* in hand usage owing to specialized training may manifest in everyday hand movement. Repeated use for many years forms stereotypic patterns of muscle coordination. A consistent set of modular structures in hand posture kinematic space has been found during various grasping tasks [181,182], tool use [181] and sign language [183]. These modules capture the averaged stereotypic behaviours and are likely encoded in the nervous system after repeated practice. Indeed, hand postures evoked by electrical microstimulation over cortical motor areas in rhesus macaques can be explained by linearly additive muscle synergies in voluntary reach and grasp [184]. Motor accent arises when topologies of *motor concepts* formed in one environment are misaligned to the cultural norms in another environment. As such, a person’s hand *motor concept topology* in their professions may manifest in their everyday hand usage as an ‘accent.’ While very few studies have directly examined the cultural influences on hand movement at both the sensorimotor and ecological levels, one important study showed that learned differences in hand movements among violinists and pianists manifest in the hand postures evoked by electrical stimulation of the motor cortex [106]. A variety of hand postures evoked among musicians differ from those of non-musicians (figure 2*c*), indicating that *motor concepts* formed and refined through extensive specialized practice are generalized across tasks, forming a basis for the culturally shaped *motor accents*. While the mean behaviours of hand grasps appear to be consistent across people and various tasks [181], the complexity of human hand control in everyday tasks appears to require a higher number of and more refined modules [185–187]. In a recent kinematic analysis of a wide variety of everyday grasps using more sensitive reflective marker-based motion tracking, after removing the first 20 modules (principal components), the remaining modules that account for small amounts of variability still carry meaningful structural information about specific grasps [188]. We speculate that cultural and individual-specific *motor concept topologies* are afforded by the high-dimensional sensorimotor flexibility enabling a rich repertoire and complexity of hand usage. We propose that culture, as a form of long-term extensive training through ‘communities of practice,’ would follow the same neuromechanical principles as expertise training, leading to observable *motor accents* exhibited in common everyday hand usage. Grasping forks compared with grasping chopsticks on a daily basis, for example, may form quite different stereotypic muscle coordination patterns. While the neuromechanics of culture-specific hand-usage *motor concepts* are yet to be studied using similar tools to those done among musicians, we speculate that similar patterns of results would hold when applying modular analysis and using brain stimulation to evoke hand postures.

## 4. Ethnokinesiological implication for motor training and rehabilitation

Our ethnokinesiology framework may be useful in understanding an individual’s *motor accent* or movement-related disability owing to injury or pathology, towards the development of more effective and personalized motor training approaches. Before an individual begins rehabilitation or motor training, they have an existing *motor concept topology* (impaired or unimpaired) based on their physical and cultural experiences. Disability is defined by the sociocultural context in which it is *perceived*, and in relation to the ‘standard’ held, in the specific environment where a person with a disability participates in activities of daily living [189]. Thus, sociocultural contexts are intricately interwoven in the notion of disability, and the importance of social determinants of health is being increasingly recognized [1]. Clinicians closely watch patients’ movement patterns to gather key information for the diagnosis of orthopaedic, neurological, and psychiatric disorders affecting motor function [190–192]. Typically in rehabilitation, measurement of baseline motor function or disability is conducted, focusing on the *sensorimotor task loop*. For example, a physical therapy clinician may evaluate sensorimotor impairments (e.g. Fugl–Meyer [193] score, or manual muscle strength of specific muscle groups) or overall motor functional capacity (e.g. overground gait speed, endurance during a 6-min walk test) using standardized clinical tests that may not translate to improved function in culturally relevant contexts. Our ethnokinesiological framework underscores the importance of measuring and accounting for factors within the *ecological task loop*. In some clinical traditions [46,194], occupational and physical therapists, speech pathologists and other healthcare clinicians try to obtain information about functional activity limitations *perceived* by the patient in real-world or community settings, in the context of interactions of the sensorimotor system with the physical environment (e.g. crossing a busy intersection safely, balance confidence scale for everyday tasks, stepping over obstacles). However, despite their importance, the interactions of the motor system with the social and cultural environment often remain poorly measured and understood. Notably, sociocultural and environmental factors that formulate the ecological loop are often challenging to track clinically and usually missing in conventional datasets or movement analysis. Emerging rehabilitation research is showing a mismatch between outcome measures of motor capacity (which are largely in the *sensorimotor task loop*) versus outcome measures of motor performance (which involve the *ecological task loop* and environmental or community factors [195,196]), suggesting that there is a need for more comprehensive and multi-modal measurement systems to characterize individual-specific *motor accents*, as supported by our ethnokinesiological framework, which in turn can help to maximize restoration of community participation and quality of life for people with disabilities.

In clinical practice, response to treatments is not only influenced by musculoskeletal injury (e.g. ankle sprain) or neural lesion (e.g. neuroanatomy of the stroke lesion), but also by the individual’s environmental and cultural background. *Experience-dependent neural plasticity* emanates from interactions of an individual’s sensorimotor system with environmental perturbations [39,197,198]. Repetitive training or rehabilitation may cause a reshaping of these *motor concept topologies*. As indicated in figure 1, the *motor concept* comprises not only the movement-related neuromechanics (neural circuits generating muscle activation, which in turn generate movement kinematics and kinetics), but equally importantly, sensory feedback and ecological consequences. During motor practice to learn a new task, all of these components of a *motor concept* may undergo changes, and the interactions among them change as well. An individual learning to play a musical instrument may aim to move with specific postures and movement styles, based on socio-cultural factors. In the case of a tennis player learning to play badminton, there may need to be a modification of the underlying muscle synergies and joint coordination along with incorporation of novel types of feedback about body and upper limb positions in relation to the racket and shuttle, as well as learning to respond to novel ecological consequences from the opposing player’s moves and verbal communication from coaches and other players on the team. Such learning might even require the unlearning of certain *motor concepts* and relearning of new ones [39,199–202]. Indeed, fractionation and merging of muscle synergies are observed in the development and training of runners [150]. Perhaps the formation of new *motor concepts* corresponds with the ‘aha’ moments we all experience when learning new motor skills. As another example, people in India were found to have a greater range of motion in their hip, knee and ankle than the Western standards in the current prosthetic limb design, possibly owing to their everyday lifestyle involving more activities such as kneeling, squatting and sitting cross-legged [203]. These neuromechanical differences caused by cultural factors influencing how we move should influence and be accounted for during the design of rehabilitation, prostheses, artificial joints for knee or hip replacement and assistive exoskeletons, but this is often not done [204,205]. Interactions between the sensorimotor and ecological loops can drive inter-individual variability in *motor concepts* at baseline, which can influence predisposition for movement-related injuries as well as response to motor training for performance enhancement and rehabilitation. There is a need to understand how culturally influenced movement experience, history, and previous training affect the severity with which an individual is affected by disease processes and how they respond to rehabilitation, which would have important implications for (p)rehabilitation.

In motor impairments, *motor concepts* and *topologies* may be altered by both pathology in the *sensorimotor task loop* and interactions in the *ecological task loop*, which may inform clinical outcomes. Beyond the variability in gait motor patterns across healthy individuals, as described above, there are also impairments in motor patterns that are prevalent in certain neurological diseases and impairments such as stroke and Parkinson’s disease. However, in both stroke and Parkinson’s disease, it is also widely recognized that there is vast heterogeneity in how motor, as well as sensory and cognitive impairments, present clinically, such that each individual may exhibit unique clusters of symptoms requiring personalized intervention [206,207]. In contrast to dancers who exhibit a greater number of muscle synergies during normal overground walking, individuals with post-stroke hemiplegia exhibit fewer gait muscle synergies the more slowly they walk [41,208]. The more a *motor concept,* as reflected in muscle synergies, generalizes across behaviours, such as balance and walking in both stroke and Parkinson’s disease, the better their motor ability [151,209]. However, post-stroke individuals with the same preferred gait speed may have different muscle synergy deficits resulting in diverse inter-joint coordination deficits [41,210] (figure 2*b*), likely requiring different rehabilitation approaches [41,211,212]. A similar loss of *sensorimotor task* control reflected in merged muscle synergies is also observed in upper limb control [87,213,214], but unrelated to neural lesions [214]. This loss of *sensorimotor task* control owing to the neural injury thus contributes to reduced motor function, yet it is unclear whether some individuals retain better motor function because they started with more complex *motor concept topologies* owing to their prior motor training or cultural environments. During motor retraining or rehabilitation, where the goal is to sculpt *motor concepts* [41,151] by leveraging neuroplasticity, an intervention that only targets either the *sensorimotor* or *ecological task loop* but not both will have limited efficacy, as well as influencing generalization to non-trained tasks (e.g. treadmill training in a physical therapy clinic carrying over to overground real-world gait, grasping a door handle during occupational therapy generalizing to other real-world upper limb tasks). Indeed, after participation in AdapTango [151], individuals with Parkinson’s disease not only improved their clinical balance scores [215], but their muscle synergies became more consistent. Thus, there is a need to account for complex intersections between movement variability induced by time-varying neuromechanical-, age-, or experience-related factors, disease-related heterogeneity and individual life history or socio-cultural environmental factors during motor retraining. A unified ethnokinesiological approach to motor training has the potential to overcome current challenges related to seemingly similar neural lesion anatomy or musculoskeletal impairments leading to variable movement impairment profiles, and the quandary of high inter-individual variability in treatment response, which merit more investigation in future studies.

Our model also predicts that without proper feedback of *ecological consequences* from one’s environment, *motor concepts* would not be properly developed and *motor concept topology* would be skewed. This prediction implies that providing proper environments and accurate *ecological feedback* in development, training, and rehabilitation is crucial. For instance, repetitive stereotypic movements are part of normal development, exercising the *sensorimotor task loop* without ecological referents. However, if the information stream from the *ecological task loop* is interrupted during development, whether through inattention/de-prioritization of the information (as possibly in the case of autism spectrum disorder) or sensory impairment (as in the case of congenital blindness), the stereotypic movements may not develop appropriately, suggesting a principled account for the repetitive motor stereotypies that appear across both autistic and blind children [216,217]. Echolalia, which is often treated as a separate indicator of autism spectrum disorders and not generally associated with movement deficits, could be seen as just another type of repetitive motor stereotypy that is associated with speech sounds [115]. Thus, having a unified model for evaluating and treating speech and other motor behaviours can help to open up novel future rehabilitation strategies.

## 5. Moving forward in ethnokinesiology

Our theoretical framework for ethnokinesiology provides a ground-level structure for understanding and studying how cultural differences in movement may be mechanistically embodied in the nervous system. The model both dissociates the *sensorimotor* and *ecological task loops*, and addresses how interactions between the two enable the formation of *motor concept topologies* that shape how the nervous system constructs movements based on both physical and sociocultural environmental factors. The framework is intentionally painted in broad strokes and may be useful in providing a concrete way to augment the many existing approaches and theories within and across areas of motor control, enabling research collaboration by providing researchers with a way to identify and interpret important sociocultural differences in movement. Here, we lay out a few major challenges in the study of cultural differences in movement.

Ethnokinesiology will need both good datasets of the biological systems and mixed-methods analyses of the rich cultural and social settings involved in human movement. Modern, quantitative approaches to *movement ethnography* will require careful collection of each individual’s sensorimotor experience and cultural environment. Recent advancements in wearable devices [218–221], markerless video-based motion capture technology [222,223] and machine learning approaches [42,43] allow us to capture and analyse large amounts of motion and physiology data across diverse populations. With the measurement of human movement ‘in the wild,’ the role of culture, environment, and practice in human movement across populations can move from descriptive to mechanistic and can span multiple sociocultural domains. The need for greater quantity, quality and diversity of data at this intersection has become urgent with the dramatic growth in AI-driven speech- and movement-based biomarkers of disease [224–230]. Significant challenges, however, still exist in many areas, such as accurate motion capture that can meet the needs of movement science [231]. Moreover, data collection ‘in the wild’ must be coupled with more controlled tasks that can serve as a point of comparison to reveal *motor accents* across groups when performing a standardized task. Perhaps an even greater challenge will be to record meaningful cultural differences in movement that can stand out above the variety of biological and physiological factors affecting movement [156,232,233]. As such, studies may first focus on distinct cultural subgroups with specific movement practices, where well-defined cultural influences can be identified and quantified. Quantification of rich personal background information will aid the mechanistic study of individual variations in movement. Data such as activity history for dancers, athletes and musicians and detailed documentation of healthcare information, such as the medical records and SOAP (subjective, objective, assessment and plan) notes [234] for patients, will be necessary.

The continued development of theories and models that explain cultural differences in movement will be critical to both interpreting large datasets, and implementing culturally informed training and rehabilitation approaches. Importantly, we emphasize that simply collecting a large quantity of movement kinematics/kinetics data and generating probabilistic descriptions of these kinematics/kinetics are not enough [235]. How can we identify *motor concepts* that are not strictly defined by the need to precisely articulate certain speech sounds, or by the need to accomplish the stringent biomechanical challenges shaping *motor concepts* in walking? Theoretically, we lack principles regarding interactions between the *sensorimotor loop* and socio-cultural factors in the *ecological loop* that shape and form *motor concepts* across a variety of contexts relevant to cultural differences in movement, for example: (i) *imitation* of others’ movements, i.e., the desire to autogenerate or recreate the sensory signals that the learner picks up from observing others, which may involve error-based learning by building and calibrating our internal models through observations and generations of new movements; a desire to conform may often, but not always, be at play in imitation; (ii) *training* in a cultural movement practice, which is often constrained by a set of implicit or explicit rules agreed-upon by a community, and by interactions with culturally relevant objects; (iii) *intention* to achieve some desired sociocultural–ecological task that leads to *ecological consequences*; and (iv) *transfer* of existing *motor concepts* to a new learning context when forming new *motor concepts*. While each has its distinct role in selecting and sculping *sensorimotor tasks* in forming *motor concepts* corresponding to particular *ecological tasks*, they also interact. For example, participation in training may involve aspects of imitation, intention and transfer.

Robotic [93] and computational biophysical modelling [236,237] approaches may be critical in showing how *motor accents* are maintained across different motor tasks [238] and in understanding the interplay between learning new *motor concepts* and potential sensorimotor or ecological costs to exploration once the task goals are imposed [239]. Data-driven methods may also aid in identifying hierarchies in the structure of movements [99] that may reveal *motor concepts*. One major challenge in applying probabilistic or deep-learning models to human *motor accents* is to embed mechanistic models of neuromechanics, which impose important constraints on behaviours. Although beyond the scope of the present work, the conceptual framework for ethnokinesiology may provide insight and a foundation for understanding how other biological factors may affect an individual’s movement, including (but not limited to) biological sex, age, physical morphology, illness, stress or fatigue. At the core of all of these questions is *understanding the sensorimotor and other biological mechanisms that enable movements that generate ecologically relevant physical and social interactions in specific cultural environments*. Only with a clear mechanistic understanding of individual and cultural differences in movement can we design appropriate personalized approaches to rehabilitation [240,241].

The ethnokinesiology framework provides a starting point that aims to facilitate collaboration across diverse researchers. Our goal is not to create an entirely new field, but rather to encourage increased communication and a principled widening of the scope of research sub-fields investigating movement. A shared conceptual model can facilitate communication by providing a common vocabulary. A concerted effort will further require good theories and models of the neuromechanics of complex human movement that intersect knowledge of biology, physiology, neuroscience, cognition, motor control, and sociological information about various ethnic groups and sub-cultures, across a wide range of body movements. Notably, there is much work ahead in using correct terminologies to distinguish biological from cultural factors (e.g. sex versus gender or sexual orientation, genetic diversity versus race or ethnicity, hearing or deaf versus Deaf, etc.) that are often conflated in movement studies (e.g. [242,243]). We hope that, over time and through rigorous studies, this framework will help facilitate an improved mechanistic understanding of how physical and sociocultural environmental factors influence individuals’ movement, and how these factors interact with biological factors affecting movement in health and disease.

## Data Availability

This article has no additional data.
